# Sequential Extraction Results in Improved Proteome Profiling of Medicinal Plant *Pinellia ternata* Tubers, Which Contain Large Amounts of High-Abundance Proteins

**DOI:** 10.1371/journal.pone.0050497

**Published:** 2012-11-20

**Authors:** XiaoLin Wu, ErHui Xiong, SuFang An, FangPing Gong, Wei Wang

**Affiliations:** Key Laboratory of Physiological Ecology and Genetic Improvement of Food Crops in Henan Province, College of Life Science, Henan Agricultural University, Zhengzhou, China; Lawrence Berkeley National Laboratory, United States of America

## Abstract

*Pinellia ternata* tuber is one of the well-known Chinese traditional medicines. In order to understand the pharmacological properties of tuber proteins, it is necessary to perform proteome analysis of *P. ternata* tubers. However, a few high-abundance proteins (HAPs), mainly mannose-binding lectin (agglutinin), exist in aggregates of various sizes in the tubers and seriously interfere with proteome profiling by two-dimensional electrophoresis (2-DE). Therefore, selective depletion of these HAPs is a prerequisite for enhanced proteome analysis of *P. ternata* tubers. Based on differential protein solubility, we developed a novel protocol involving two sequential extractions for depletion of some HAPs and prefractionation of tuber proteins prior to 2-DE. The first extraction using 10% acetic acid selectively extracted acid-soluble HAPs and the second extraction using the SDS-containing buffer extracted remaining acid-insoluble proteins. After application of the protocol, 2-DE profiles of *P. ternata* tuber proteins were greatly improved and more protein spots were detected, especially low-abundance proteins. Moreover, the subunit composition of *P. ternata* lectin was analyzed by electrophoresis. Native lectin consists of two hydrogen-bonded subunits (11 kDa and 25 kDa) and the 11 kDa subunit was a glycoprotein. Subsequently, major HAPs in the tubers were analyzed by mass spectrometry, with nine protein spots being identified as lectin isoforms. The methodology was easy to perform and required no specialized apparatus. It would be useful for proteome analysis of other tuber plants of Araceae.

## Introduction

*Pinellia ternata*, a member of the Araceae family, is a perennial monocot herb native to Asia and also grows as an invasive weed in parts of North America [1]. Its tuber has specific pharmacological properties such as anti-emesis, anti-tussis, anti-obesity, anti-inflammatory etc [1]–[3]. For thousands of years, *P. ternata* tubers have been used as an important constituent in traditional Chinese medicines. Today *P. ternata* is officially listed in the Chinese pharmacopoeia. However, the mechanism of specific pharmacological effects of *P. ternata* tubers remains poorly understood, due to its complex constituents [3], [4]. It is expected that proteome analysis of *P. ternata* tubers will provide much information on protein constituents and help to elucidate the pharmacological properties of tuber proteins of interest.

*P. ternata* tubers contain a high concentration of proteins, typically 3–7% of fresh weight. During the last few decades, mannose-binding lectin (agglutinin) in *P. ternata* tubers has received much interest and intensive research. The major tuber lectin is a tetrameric protein of 40–50 kDa composed of two polypeptide chains that are slightly different in size and very different in isoelectric point (pI) [5]. Its gene constitutively expresses in various tissues including tuber, leaf, stem and inflorescence [6]. Another characterized *P. ternata* lectin is a 6 kDa glycoprotein, with contents from 5.75 to 8.30% in the tubers [7]. In addition, *P. ternata* tuber also contains a 25 kDa lectin [8]. *P. ternata* lectin exhibits various pharmacological and biological activities including termination of pregnancy, antitumor activity and insecticidal effects [7], [9], [10]. Recently, monocot lectins have become an important tool in plant protection and biotechnology, because their genes confer plant resistance against sucking insects and nematodes. To better understand the regulation mechanism of lectin biosynthesis, it is useful to perform proteome analysis of *P. ternata* tubers.

An examination of the scientific literature indicated that no successful proteome profiling of *P. ternata* tubers has yet been reported. In preliminary experiments, we found that a few high-abundance proteins (HAPs) existed in large amounts in the tubers and seriously interfered with proteome profiling of *P. ternata* tubers. For proteome analyses, a small sample volume (about 10–300 μl) is typically used, consequently a large percentage of expressed proteins are not present in sufficient quantities to be detected, especially low-abundance proteins (LAPs). Many studies showed that LAPs can be enriched from crude extracts by biochemical approaches such as selective fractionation, chromatography, preparative isoelectric focusing (IEF) or by a combination of these approaches [11]–[14]; however, most of the available approaches are time-consuming and require expensive instrumentation. Therefore, the ability to selectively remove or deplete HAPs from biological samples is increasingly important in proteome analyses.

As the first step in our proteome analysis strategy, we developed a sequential extraction protocol to deplete lectin and prefractionate proteins of *P. ternata* tubers. Based on differential protein solubility, the protocol involved acid extraction and SDS extraction. The protocol allowed for the enhanced *P. ternata* tubers proteome analysis and detection of more protein spots, especially LAPs. The recovery and solubilization of sequentially extracted proteins were also optimized. Furthermore, the subunit composition of *P. ternata* lectin and major HAPs were analyzed by electrophoresis and mass spectrometry. The methodology would be useful for the proteomic analysis of other tuber species of Araceae.

## Materials and Methods

### Preparation of acetone tuber powder

Fresh tubers (1.2–1.5 cm in diameter) of wild *Pinellia ternata* were peeled and cut into pieces. An aliquot (0.1–0.2 g) of the tubers was homogenized in a mortar in 2 ml cold acetone plus 0.07% 2-mecaptoethanal. The homogenate was transferred into microtubes and centrifuged at 15,000 g for 10 min (4°C). The supernatant was discarded. The pellet was rinsed twice with cold acetone plus 0.07% 2-mecaptoethanal and centrifuged as above. The final pellet was air-dried and used as starting material for protein extraction.

### Total protein extraction from P. ternata tubers

For comparison with sequential extraction, total proteins were extracted from *P. ternata* tuber tissue powder using SDS sample buffer and purified with phenol extraction, as described below. Finally, total tuber proteins were dissolved in SDS sample buffer or 2-DE rehydration buffer.

### Sequential extraction of P. ternata tuber proteins

The sequential extraction protocol consisted of 10% acetic acid extraction and SDS extraction. The protocol could be scaled up to use in larger centrifuge tubes. Unless otherwise indicated, all steps below were performed at room temperature.

#### Acid extraction

About 0.015 g of the dry tuber powder was extracted in 1.5 ml 10% acetic acid by grinding in a mortar and then transferred into microtubes. Crude extract was centrifuged at 15,000 g for 10 min. The supernatant was taken to new tubes. The precipitate was extracted with 1 ml 10% acetic acid twice by shaking for 10 min and centrifuged as above. All the supernatants (acid extract) from 3 extractions were pooled, and acid-soluble proteins in acid extracts were precipitated with 5 volumes of acetone for 2 h (−20°C).

#### SDS extraction

The precipitate after acetic acid extraction was washed twice with cold acetone to remove residual acetic acid and then extracted in 1.5 ml the SDS buffer consisting of 0.5 M Tris-HCl, pH 8.8, 2% SDS, 20 mM dithiothreitol (DTT), and 0.001% bromophenol blue. The mixture was heated at 70°C for 10 min to facilitate protein extraction and then centrifuged as above. The SDS extraction was repeated once more, if applicable. The supernatant (SDS extract) was added with equal volume of phenol (pH 8.0) [15] and thoroughly mixed by shaking for 5 min. Phase separation was achieved by centrifugation as above. The phenol phase (containing proteins) was transferred to new microtubes and precipitated with 5 volumes of methanol containing 0.1 M ammonium acetate for 2 h (−20°C).

#### Protein recovery and solubilization

Protein pellets from acid extract and SDS extract were recovered by centrifugation at 15,000 g for 5 min (4°C), washed with cold acetone twice, air-dried and dissolved in SDS sample buffer [16] or 2-DE rehydration buffer (7 M urea, 2 M thiourea, 4% CHAPS, 2% IPG buffer, 20 mM DTT), respectively.

### Protein quantification

Protein was quantified by Bradford protein assay [16] with bovine serum albumin as a standard.

### One-dimensional electrophoresis (1-DE)

Native PAGE (6% gel) and SDS-PAGE (4.75% stacking gel and 13.5% resolving gel) were performed according to Laemmli [17]. Tricine-SDS-PAGE was performed as described previously [18]. After electrophoresis, proteins in gels were visualized with Coomassie brilliant blue R (CBB).

### Detection of glycoproteins

Total proteins of *P. t*ernata tubers were subjected to SDS-PAGE and stained using commercial glycoprotein detection kit (BSP035, Sangon Biotech, Shanghai, China) according to the users' guidelines. Glycoproteins emerged as a blue color after Alcian blue staining of the gel [19].

### Two-dimensional electrophoresis (2-DE)

Proteins in 2-DE rehydration buffer were loaded onto an 11-cm linear pH 4–7 strip via passive rehydration overnight [15]. IEF was performed with the Ettan III system (GE Healthcare, USA) at 250 V for 1 h, 1,000 V for 1 h and 8,000 V for 10 h (20°C). Focused strips were equilibrated in Buffer I (0.1 M Tris–HCl, pH 8.8, 4% SDS, 6 M urea, 30% glycerol, 0.1 M DTT) for 15 min and then for another 15 min in Buffer II (its composition was the same as Buffer I, but with 0.25 M iodoacetamide replacement of DTT). SDS-PAGE was run in 13.5% gel (20×15×0.1 cm), with 0.2% SDS in the gel and the running buffer. The gels were stained with 0.1% CBB and destained in 10% acetic acid. 2-DE profiles were analyzed by PDQuest 2-DE analysis software (Version 6.2, Bio-Rad, UK). Protein spots of interest were subjected to MALDI-TOF MS/MS analysis for protein identification.

### Mass spectrometry and protein identification

Protein spots of interest were excised from 2-DE gels and processed for trypsin digestion [20]. Proteins were reduced (10 mM DTT), alkylated (50 mM iodoacetic acid) and then digested with 10 μg/μl trypsin for 16 h at 37°C in 50 mM ammonium bicarbonate. The supernatants were vacuum-dried and dissolved in 10 μl 0.1% trifluoroacetic acid and 0.5 μl added onto a matrix consisting of 0.5 μl of 5 mg/ml 2–5-dihydroxybenzoic acid in water: acetonitrile (2∶1). The digested fragments were analyzed on a MALDI-TOF/TOF analyzer (ultraflex III, Bruker, Germany). MS/MS spectra were acquired in the positive-ion mode and automatically submitted to Mascot 2.2 (http://www.matrixscience.com, Matrix Science) for peptide mass finger printings against the NCBInr 20120922 database (20543454 sequences, http://www.ncbi.nlm.nih.gov/). The taxonomy was Viridiplantae (green plants) (1093036 sequences). The search parameters were as follows: type of search: MS/MS Ion Search; enzyme: trypsin; fixed modifications: carbamidomethyl (C); variable modifications: acetyl (Protein N-term), Oxidation (M); mass values: monoisotopic; protein mass: unrestricted; peptide mass tolerance: ±120 ppm; fragment mass tolerance: ±0.4 Da; max missed cleavages: 1; instrument type: MALDI-TOF-TOF. Only significant scores defined by Mascot probability analysis greater than “identity” were considered for assigning protein identity. All of the positive protein identification scores were significant (P<0.05, score>60). Theoretical Mr and pI of identified proteins were predicted at http://www.expasy.ch/tools/pI_tools.html.

## Results

In the present study, acetone powder of *P. ternata* tubers was used as starting material for protein extraction. This practice removed most of pigments, phenolic substances and other secondary metabolites and minimized protein degradation occurring during protein extraction. Besides, it reduced volume of fresh tubers and facilitated manipulation in microtubes.

### Total protein extraction and electrophoretic separation of P. ternata tubers

**Figure 1:**
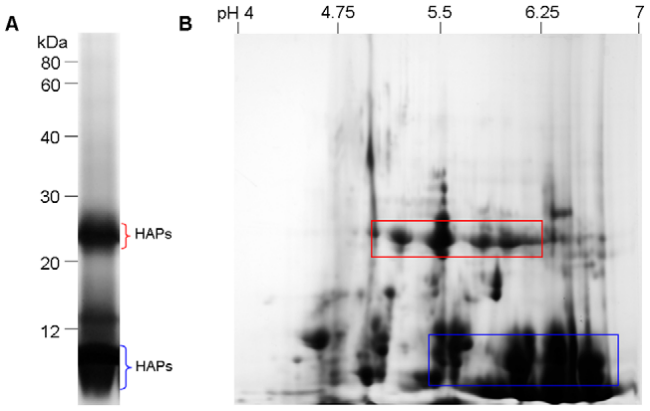
Protein profiles of total proteins of *Pinellia ternata* tubers separated by SDS-PAGE and 2-DE. Total protein was extracted from acetone tissue powder of *P. ternata* tubers with SDS-containing buffer and purified by phenol extraction. Protein loads were 30 μg (**A**) and 400 μg (**B**), respectively. Gels were stained with CBB R. HAPs in tuber extract were indicated. Rectangles: indicating the mask effect of HAPS on other protein spots (**B**).

In preliminary experiments, total proteins of *P. ternata* tubers were extracted by conventional SDS extraction and purified by phenol extraction. SDS-PAGE showed that in tuber crude extract, two prominent bands (HAPs) at 11 kDa and 25 kDa, respectively, existed in high-abundance, while most other bands were present in low-abundance and poorly resolved (Figure 1, A). These HAPs were estimated to take up about 90% of total tuber proteins. Based on relative molecular mass (Mr), the 25 kDa band may represent the extensively characterized lectin in *P. ternata* tubers [6], [8]; the two prominent bands were probably subunits of native 40–50 kDa tetrameric aggregate of tuber lectin [5].

2-DE analysis revealed that the HAPs existed in many isoforms emerging in a smeared pattern and masking other proteins in a large area (indicated by rectangles; Figure 1, B). In fact, few distinct spots were well resolved in the 2-DE gel. Thus, HAPs themselves were badly resolved and also hindered the separation of other proteins. The 2-DE profile of *P. ternata* tuber proteins was seriously damaged by these HAPs due to their masking effect. For enhanced proteome analysis these HAPs needed to be depleted, at least partly, from tuber proteins prior to 2-DE.

### Sequential extraction and electrophoretic separation of P. ternata tuber proteins

**Figure 2:**
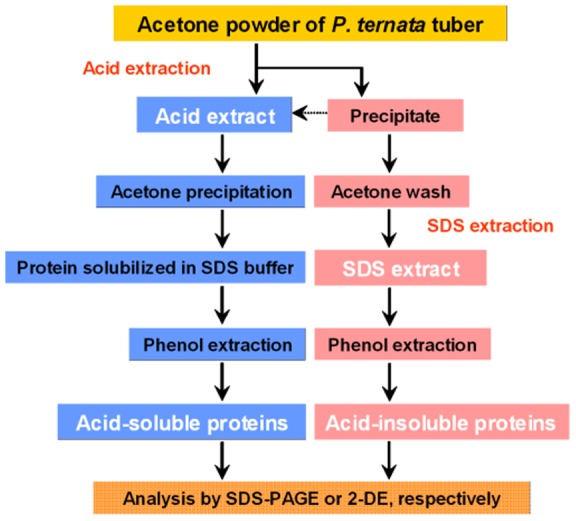
Workflow for sequential protein extraction protocol. Acetone tissue powder of *Pinellia ternata* tubers was sequentially extracted by 10% acetic acid, followed by SDS-containing buffer.

After much effort, we developed an efficient sequential extraction protocol to deplete HAPs and prefractionate *P. ternata* tuber proteins. The protocol involved an initial extraction by acetic acid to selectively extract acid-soluble HAPs and the second extraction by SDS-containing buffer to extract remaining acid-insoluble proteins. The workflow of the sequential extraction protocol is shown in Figure 2.

**Figure 3:**
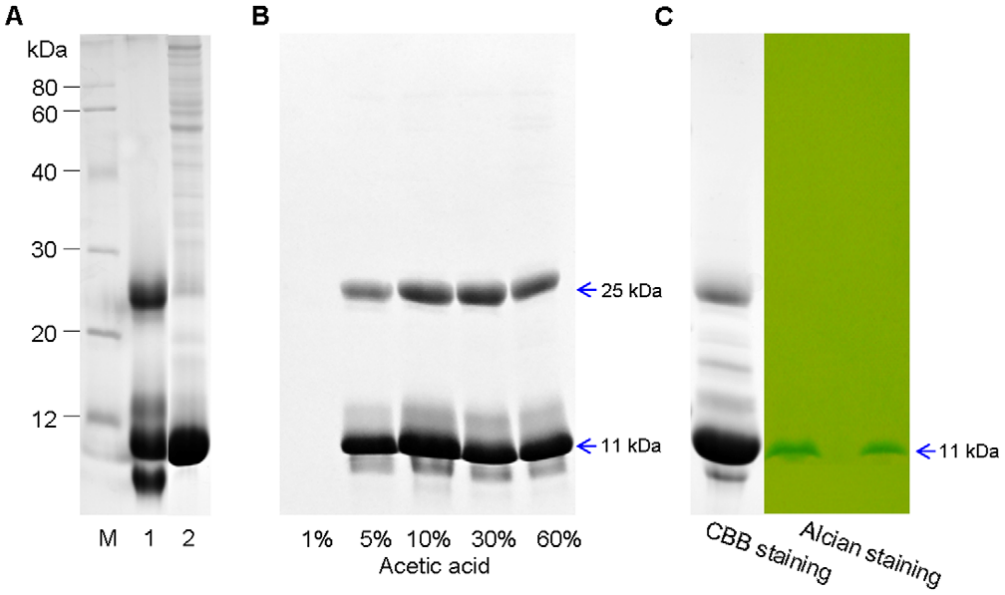
Sequential protein extraction improved the SDS-PAGE profiles of *P. ternata* tuber proteins. **A**, equal amounts of proteins (25 μg) from each extract were separated by SDS-PAGE. **B**, depletion effect of different concentrations of acetic acid. **C**, detection of glycoproteins in *P. ternata* tubers. Total tuber proteins were resolved by SDS-PAGE and visualized by CBB (protein load 25 μg) and Alcian stain (protein load 10 μg), respectively. Only the 11 kDa band was stained by Alcian staining, indicating a glycoprotein.

Electrophoretic comparisons revealed qualitative differences in protein profiles between the acid extract and the SDS extract (Figure 3, A). The major 25 kDa HAPs in *P. ternata* tubers were selectively extracted and enriched by 10% acetic acid, while the 11 kDa HAPs were only partly extracted, indicating there existed a great difference in solubility between the 25 kDa and 11 kDa proteins. After acetic acid extraction, the precipitate was rinsed twice with acetone to remove residual acetic acid and extracted with the SDS-containing buffer (0.5 M Tris-HCl, pH 8.8, 2% SDS, 20 mM DTT), which had adequate pH and ion strength to buffer residual acetic acid. Remarkably, 11 kDa HAPs were enriched in the SDS extract, and more LAPs became obvious, especially in the high Mr region; no visible 25 kDa HAPs were observed, indicating depletion by 3 continuous acid extractions. The profiles of acid-soluble proteins and acid-insoluble proteins were much better than that of total tuber proteins (Figure 1, A).

In addition, the extraction efficiency of 1%, 5%, 10%, 30% and 60% acetic acid were compared. 10% acetic acid was found to be enough for efficiently extraction of 25 kDa HAPs (Figure 3, B). Various aqueous buffers, salts and basic solutions were examined for sequential extraction, but their efficiencies were much poorer than acetic acid, especially the 25 kDa HAPs which were not solubilized in 0.1 M NaOH (data not shown). Glycosylation analysis showed that the 11 kDa lectin was a glycoprotein, but the 25 kDa proteins were not (Figure 3, C). It may explain their difference in solubility in acetic acid.

**Figure 4:**
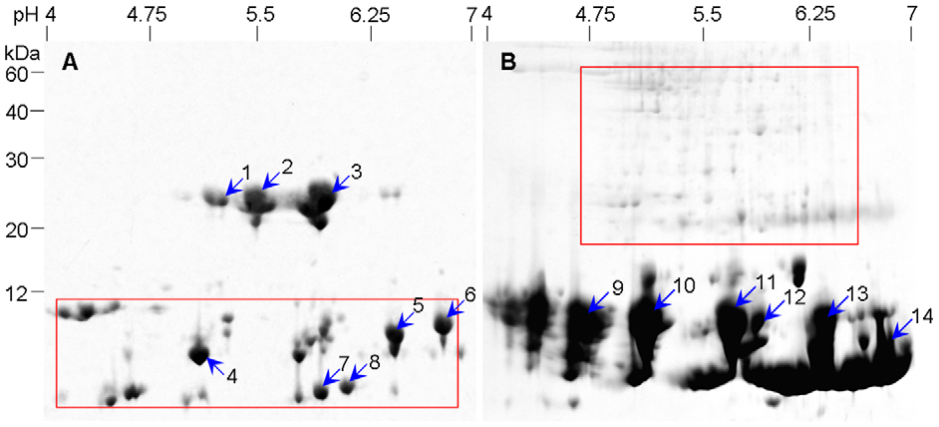
Sequential protein extraction improving 2-DE profiles of *P. ternata* tuber proteins. **A**, acid extract; protein load 200 μg. **B**, SDS extract, protein load 400 μg. Rectangles: used to highlight different protein patterns in two gels, indication more new protein spots emerged. Shown were representative CBB-stained gels.

Depletion of HAPs was expected to improve 2-DE protein profiles by enabling detection of protein spots that were previously masked and by increasing protein loads. To further characterize tuber proteins, both acid-soluble and acid-insoluble proteins were subjected to 2-DE. For acid-soluble proteins, less protein loads were applied due to fewer kinds of proteins in the acid extract (see Figure 3, A). Several isoforms of 25 kDa HAPs in the acid extract were separated from each other and many spots (indicated by a rectangle) previously masked by 11 kDa lectin were clearly resolved (Figure 4, A). In the SDS extract, the most prominent feature was an increase of LAPs spots (indicated by rectangle) in the high Mr region and the 11 kDa lectin isoforms overwhelmingly occupied in the low Mr region (Figure 4, B). By sequential extraction, acid-soluble proteins (mainly 25 kDa HAPs) and other acid-insoluble proteins such as 11 kDa lectin were separated into different fractions, with substantial spots exclusive to the acid extract or the SDS extract. As a result, sequential extraction reduced the complexity of *P. ternata* tuber proteome and highly improved protein profiling of acid-soluble proteins and acid-insoluble proteins compared to unfractionated total proteins. As an aside, a 13.5% gel in second dimension resolved proteins better than 12.5% gel, when applying 2-DE analysis of *P. ternata* tuber proteins, especially low-weight proteins (data not shown).

A common way to evaluate the sequential extraction fractionation was to compare the number of distinct proteins detected in each fraction. In CBB-stained 2-DE gels, about 68 spots were detected in acid extract, 121 spots in SDS extract, and in total 189 spots were detected. There was little overlap between the two extracts. By comparison, few spots were well resolved in the unfractionated total protein extract (Figure 1, B) of *P. ternata* tubers.

### Subunit composition and MS/MS identification of HAPs in P. ternata tubers

**Figure 5:**
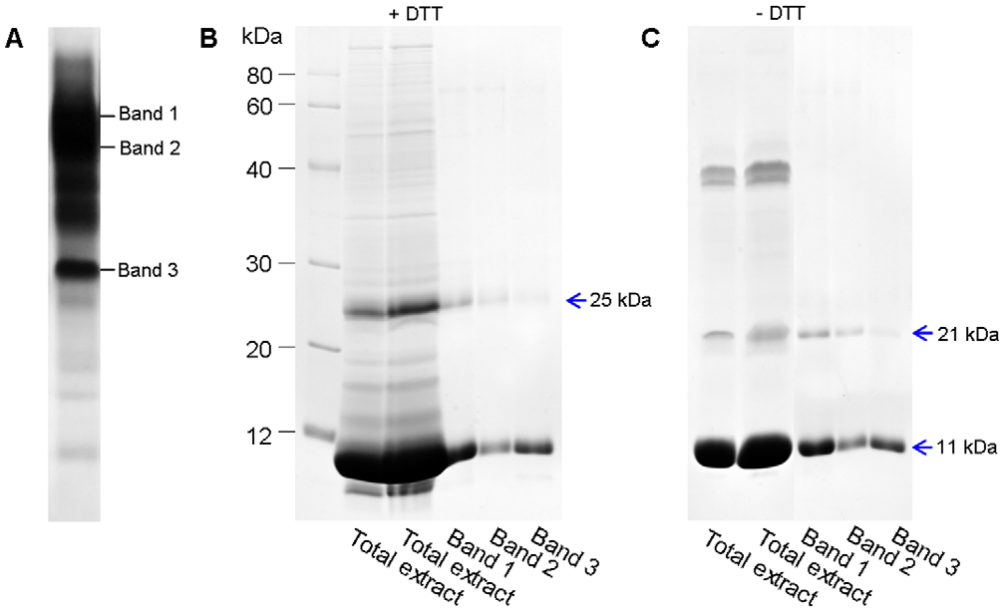
Identification of subunit compositions of lectins in *P. ternata* tubers. **A**, native gel, total tuber proteins. **B**, total tuber proteins and purified Bands 1–3 were resolved by SDS-PAGE under reducing conditions (with DTT). **C**, total tuber proteins and purified Bands 1–3 were resolved by SDS-PAGE under reducing conditions (without DTT).

The profile of native gel revealed the existence of three major bands (designed as Band 1, 2 and 3; Figure 5, A) in the crude extract of *P. ternata* tubers, whereas SDS-PAGE only resolved two major bands (11 kDa and 25 kDa; Figure 1, A). We subsequently sought to examine the relationship between these bands. The three major bands from the native gel were recovered and analyzed by SDS-PAGE under non-reducing or reducing conditions, respectively. As a result, Band 1, 2 and 3 were dissociated into two identical subunits: one small band (11 kDa) in abundance and another large band (21 kDa in the absence of DTT or 25 kDa in the presence of DTT) in less abundance. Therefore, the three major bands from the native gel belong to lectin aggregates of different sizes, and native lectin consisted of two subunits (11 kDa and 25 kDa) which were linked by hydrogen bonds.

Interestingly, the apparent size of large subunit of lectin was greater in non-reducing conditions than in non-reducing conditions. Probably, this phenomenon was due to the existence of intra-chain disulfide-bonds, which made the molecular structure of the large subunit more compact even in denaturing conditions and exhibited a smaller size. In fact, amino acid sequences of all 7 cloned lectin genes in the NCBI database contain 4 cysteine residues which may form two potential intra-chain disulfide-bonds (Figure S1).

Finally, the possible isoforms of *P. ternata* tuber lectin (indicated by numbers; Figure 4) were subjected to MALDI-TOF MS/MS analysis. Of the 14 protein spots selected for MS/MS analysis, 9 were successfully identified and matched to five agglutinins (NCBI accession: gi|31559037, gi|374341512, gi|122912961, gi|118421163 and gi|33355625) from four species in the same family (Araceae) (Table S1). These agglutinins shared 80%–86% identity in amino acid sequence (Figure S2). Obviously, identified lectins (spots 4–6) from acid extract were different in solubility from those (spots 9–14) from SDS extract. So, the protocol reported here was compatible with MS analysis.

## Discussion

2-DE coupled with mass spectrometry has proven to be an efficient means of proteome analysis [21]. However, successful proteomic analysis of biological samples is often hindered by HAPs, which make the detection of LAPs a major challenge [22]. Therefore, the ability to selectively deplete HAPs for enhanced detection of LAPs is increasingly important in proteomic studies of biological samples.

*P. ternata* tubers contain a high concentration of proteins, typically in the range of 3–7% of fresh weight and a few HAPs constitute roughly 90% of total tuber proteins. These HAPs greatly interfere with tuber protein separation by 2-DE. Furthermore, proteomic analysis of sufficient sample to detect LAPs in *P. ternata* tubers means excessive loads of several HAPs, which invariably mask the separation of LAPs. HAP depletion was a prerequisite for successful tuber proteome analysis. The sequential extraction protocol described here improved the quality of 2-DE gels in two ways: first, depletion of a 25 kDa and other acid-soluble proteins resulted in the improved separation of both acid-extractable and SDS-extractable proteins and the emergence of many new spots, especially LAPs (Figure 4); second, after depletion of acid-soluble proteins, the load of LAPs in SDS extract were substantially increased. In CBB-stained 2-DE gels, a total of 68 spots were detected in the acid extract and 121 spots in the SDS extract, compared to few spots well resolved in the unfractionated total protein extract of *P. ternata* tubers. It is expected that silver staining would visualize even more protein spots. So, the protocol described here provided an effective way to prefractionate *P. ternata* tuber proteins for enhanced proteome analysis.

In 2-DE protein sample preparation, sequential extraction strategies are universally exploited, most with alternations in pH and ionic strength of extraction buffers [23]. In this present study, however, the first sequential extraction step used acetic acid, in which most proteins were not dissolved in this harsh organic solvent with extremely low pH. Unexpectedly, 25 kDa HAPs could be selectively and completely depleted by 10% acetic acid, which facilitated the following SDS extraction and 2-DE separation of remaining proteins. Generally, 3 rounds of extraction using acetic acid can deplete 25 kDa HAPs. This suggested that the 11 kDa lectin was different from 25 kDa HAPs in its solubility. It has been suggested that the 25 kDa HAPs were similar in solubility with acidic-soluble storage protein (glutelin) in cereal seeds [24]. Previous studies reported that acetic acid (1–60%) is an effective solvent for protein extraction of solid tissues and purification of polypeptides [25]–[27]. The second extraction used the SDS buffer, in which 0.5 M Tris-HCl (pH 8.8) was able to neutralize the trace amount of acetic acid and maintain the pH of the extract above 7. Furthermore, bromophenol blue in the SDS buffer was a good pH indicator, which is compatible with phenol and remains blue at pH levels above 7.

It should be noted that it was difficult to obtain a better 2-DE profile of the extract containing *P. ternata* tuber lectin (Figure 4, B) compared to the 1-DE profile. This was because that 11 kDa lectin is a glycoprotein (Figure 3, C), which may result in horizontal streaking on 2-DE gels. The removal of carbohydrate moieties by chemical or enzymatic methods can alleviated the horizontal tailing of glycoproteins in 2-DE [28]. The 11 kDa lectin could be removed from the SDS extract by other approaches such as cut-off membrane technology, however, its complete removal may not be desirable because it may trap many of LAPs or other proteins, which will thus be lost and not detected. Nonetheless, protein recovery from each extraction was optimized: acid-soluble proteins were precipitated with acetone and SDS-extracted proteins were purified with a phenol extraction process; these practice allowed for the removal of non-protein substances (including polysaccharides) [15].

In the present study, the subunit composition of lectin was analyzed by electrophoresis. Our results showed that native lectin consisted of two subunits (11 kDa and 25 kDa) which were linked by hydrogen bonds and these subunits could form many lectin aggregates of different sizes. The large subunit should be the lectin of 25 kDa [8], but the ratio of the 25 kDa and the 11 kDa subunit in lectin aggregates was uncertain. It should be noted that the subunit composition of *P. ternata* lectin reported here somewhat disagrees with a previous study indicating that *P. ternata* lectin is a tetramer of 40–50 kDa composed of four polypeptide chains, each with similar Mr (11–14 kDa) but different pI [5].

In the present study, nine isoforms of lectin from *P. ternata* tubers were successfully identified by MS/MS analysis (Table S1). Five spots from acid extract (Figure 4, A) failed to be identified. Since *P. ternata* is a non-model plant species and only 128 nucleotide sequences (unverified) on *P. ternata* were retrieved from the NCBI database (search on Oct. 12, 2012): most entries are derived from microsatellites and chloroplast proteins. Similarly, in UniProtKB/Swiss-Prot, there were only 20 entries for *P. ternata* proteins. In addition, only 2985 protein sequences were retrieved for Araceae from the UniProtKB/Swiss-Prot database.

In conclusion, our study showed that sequential extraction of *P. ternata* tuber proteins before 2-DE proves to be a powerful tool for rapid and specific depletion of acid-soluble HAPs, allowing for an easier detection of other proteins, especially LAPs. Acetic acid depletion of *P. ternata* HAPs is simple, quick and requires no expensive instrumentation. The protocol allows for an enhanced *P. ternata* tuber proteome analysis and would be useful for proteome profiling of other tuber species of Araceae.

## Supporting Information

Figure S1 **Multiple sequence alignment with hierarchical clustering.** Seven sequences of *Pinellia ternata* lectin were retrieved from NCBI database and their proteins all contained 4 cysteine residues (indicated by rectangles). Alignment was according to Corpet (Nucl Acids Res, 1988, 16: 10881–10890). Consensus symbols: ! is anyone of IV, $ is anyone of LM, % is anyone of FY, # is anyone of NDQEBZ.
(TIF)

Figure S2 **Multiple sequence alignment with hierarchical clustering and homology among agglutinin from Araceae.** *Pinellia ternata* agglutinin (gi|374341512) shares high homology (80%–86% identity) with other four agglutinin sequences in Table S1. Alignment was according to Corpet (Nucl Acids Res, 1988, 16: 10881–10890). Consensus symbols: ! is anyone of IV, $ is anyone of LM, % is anyone of FY, # is anyone of NDQEBZ.
(TIF)

Table S1 **MS/MS identification of agglutinin (lectin) in** ***Pinellia ternata*** **tuber.** Notes: a, spot number referred to Figure 4. Spot 1–3, 7 and 8, failed to be identified and not included in the table. b, agglutinin is the synonym of lectin, which can bind mannose. In the present work, protein names were not normalized and kept their original names in NCBI database. c, accession in NCBI database. d, all species listed belong to the genus *Arisaema*, *Pinellia* and *Typhonium,* respectively, in the Araceae family. e, Theoretical Mr and pI of identified proteins were predicted at http://www.expasy.ch/tools/pI_tools.html.
(DOC)
