# Supplementary figures and images for: Sequential Extraction Results in Improved Proteome Profiling of Medicinal Plant Pinellia ternata Tubers, Which Contain Large Amounts of High-Abundance Proteins
Source: PLoS One. 2012 Nov 20;7(11):e50497. doi: 10.1371/journal.pone.0050497 (PMC3502364; doi:10.1371/journal.pone.0050497)

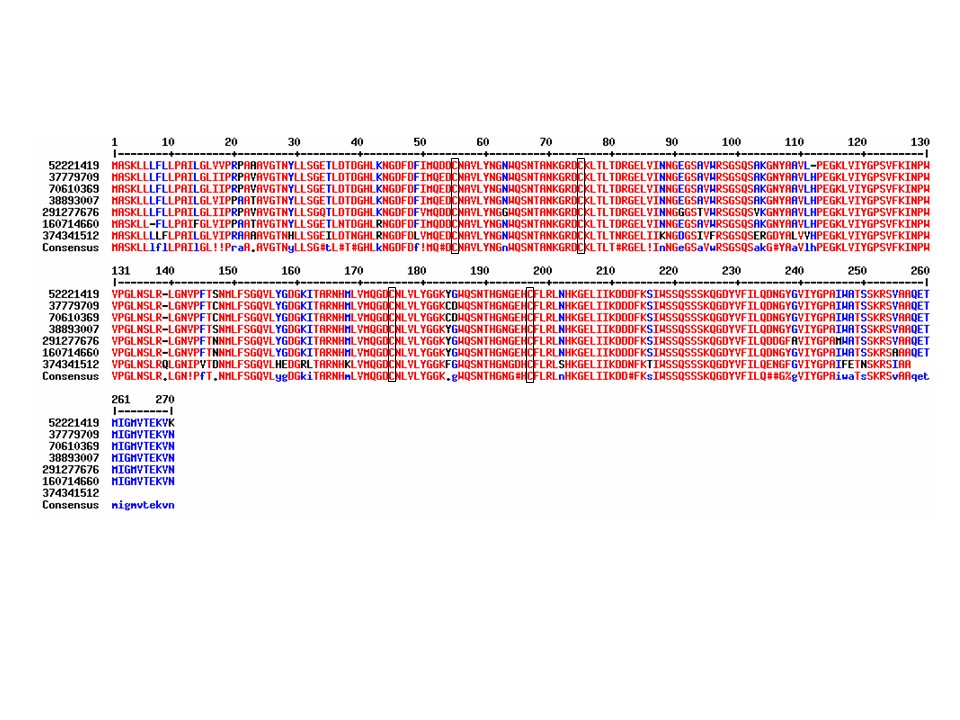

Supplement: Figure S1 — Multiple sequence alignment with hierarchical clustering. Seven sequences of Pinellia ternata lectin were retrieved from NCBI database and their proteins all contained 4 cysteine residues (indicated by rectangles). Alignment was according to Corpet (Nucl Acids Res, 1988, 16: 10881–10890). Consensus symbols: ! is anyone of IV, $ is anyone of LM, % is anyone of FY, # is anyone of NDQEBZ. (TIF) [file pone.0050497.s001.tif]

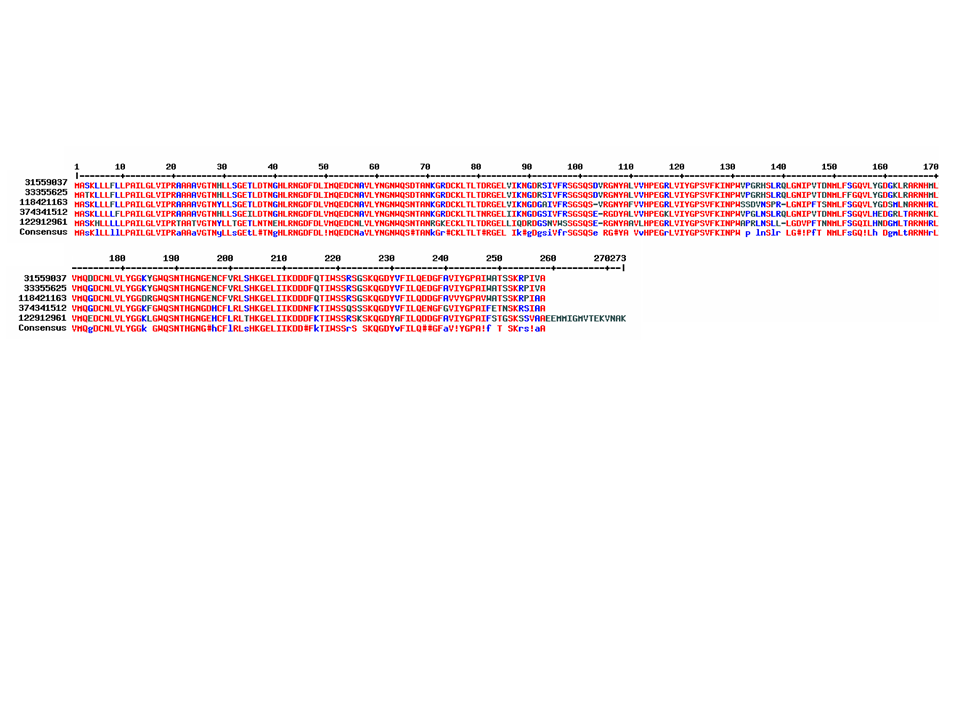

Supplement: Figure S2 — Multiple sequence alignment with hierarchical clustering and homology among agglutinin from Araceae. Pinellia ternata agglutinin (gi|374341512) shares high homology (80%–86% identity) with other four agglutinin sequences in Table S1. Alignment was according to Corpet (Nucl Acids Res, 1988, 16: 10881–10890). Consensus symbols: ! is anyone of IV, $ is anyone of LM, % is anyone of FY, # is anyone of NDQEBZ. (TIF) [file pone.0050497.s002.tif]
