# Supplementary material for: Sequential Extraction Results in Improved Proteome Profiling of Medicinal Plant Pinellia ternata Tubers, Which Contain Large Amounts of High-Abundance Proteins
Source: PLoS One. 2012 Nov 20;7(11):e50497. doi: 10.1371/journal.pone.0050497 (PMC3502364; doi:10.1371/journal.pone.0050497)
Supplement: Table S1 — MS/MS identification of agglutinin (lectin) in Pinellia ternata tuber. Notes: a, spot number referred to Figure 4. Spot 1–3, 7 and 8, failed to be identified and not included in the table. b, agglutinin is the synonym of lectin, which can bind mannose. In the present work, protein names were not normalized and kept their original names in NCBI database. c, accession in NCBI database. d, all species listed belong to the genus Arisaema, Pinellia and Typhonium, respectively, in the Araceae family. e, Theoretical Mr and pI of identified proteins were predicted at http://www.expasy.ch/tools/pI_tools.html. (DOC) [file pone.0050497.s003.doc]

| **Spot** | **Protein name** | **Accession** | **Species** | **pI/MW** | **Mascot Score** | **Matched peptides** |
| --- | --- | --- | --- | --- | --- | --- |
| 4 | Agglutinin (lectin) | gi | 31559037 | *Arisaema heterophyllum* | 7.71/28.7 | 132 | R.GNYALVVHPEGR.L  K.LTLTDRGELVIK.N |
| 5 | Agglutinin | gi | 374341512 | *Pinellia ternata* | 7.72/28.4 | 200 | K.NGDGSIVFR.S  R.SGSQSERGDYALVVHPEGK.L  K.LVIYGPSVFK.I |
| 6 | Mannose-binding tuber lectin | gi | 122912961 | *Typhonium divaricatum* | 6.24/30.2 | 96 | R.GNYAAVLHPEGR.L |
| 9 | Mannose-binding lectin | gi | 118421163 | *Pinellia cordata* | 6.95/28.2 | 37 | R.GNYAFVVHPEGR.L |
| 10 | Mannose-binding lectin AHA | gi | 33355625 | *Arisaema heterophyllum* | 8.32/28.7 | 93 | R.GNYALVVHPEGR.L |
| 11 | Agglutinin | gi | 31559037 | *Arisaema heterophyllum* | 7.71/28.7 | 90 | R.GNYALVVHPEGR.L |
| 12 | Mannose-binding tuber lectin | gi | 122912961 | *Typhonium divaricatum* | 6.24/30.2 | 47 | R.GNYAAVLHPEGR.L |
| 13 | Mannose-binding lectin | gi | 118421163 | *Pinellia cordata* | 6.95/28.2 | 98 | R.GNYAFVVHPEGR.L  R.LVIYGPSVFK.I |
| 14 | Agglutinin | gi | 31559037 | *Arisaema heterophyllum* | 7.71/28.7 | 78 | R.GNYALVVHPEGR.L  R.LVIYGPSVFK.I |

**Table S1** MALDI-TOF-TOF identification of Agglutinin (lectin) in *Pinellia ternata* tuber
